# Reaching and Grasping in Autism Spectrum Disorder: A Review of Recent Literature

**DOI:** 10.3389/fneur.2014.00006

**Published:** 2014-01-23

**Authors:** Lori-Ann R. Sacrey, Tamara Germani, Susan E. Bryson, Lonnie Zwaigenbaum

**Affiliations:** ^1^Department of Pediatrics, University of Alberta, Edmonton, AB, Canada; ^2^Departments of Pediatrics and Psychology, Dalhousie University, Halifax, NS, Canada; ^3^IWK Health Centre, Halifax, NS, Canada; ^4^Glenrose Rehabilitation Hospital, Edmonton, AB, Canada

**Keywords:** reaching, reach-to-grasp, motor planning, motor execution, movement, autism spectrum disorder, review

## Abstract

Impairments in motor functioning, which, until recently, have rarely been a primary focus in autism spectrum disorder (ASD) research, may play a key role in the early expression of biological vulnerability and be associated with key social-communication deficits. This review summarizes current knowledge of motor behavior in ASD, focusing specifically on reaching and grasping. Convergent data across the lifespan indicate that impairments to reaching and grasping emerge early in life, affect the planning and execution of motor programs, and may be impacted by additional impairments to sensory control of motor behavior. The relationship between motor impairments and diagnostic outcomes will be discussed.

## Introduction

Autism spectrum disorder (ASD) is a developmental disorder characterized by impairments in social communication and the presence of repetitive or restricted behaviors (1). ASD is one of the most prevalent forms of developmental disability internationally, with current estimates at 1 in 88 (2, 3). In his original case series, Kanner (4) described ASD primarily in relation to severe impairment in social–emotional and communication ability but also commented on several aspects of motor development: motor milestones were generally *within normal limits* and fine motor coordination was “*very skillful*,” although some patients had gross motor deficits. However, more recent reports suggest that gross and fine motor deficits are prevalent in ASD (5–10) and include impairments in basic motor control (11–13), difficulty performing skilled motor gestures (14, 15), abnormal patterns of motor learning (16, 17), and disturbances in the reach-to-grasp movement (18, 19). To date, these motor abnormalities are categorized as “associated (as opposed to core) symptoms” (8) and are thought to interfere with the development of adaptive skills (15, 20–22). Motor impairments may have primary effects on achieving independence in activities of daily living (such as holding a spoon), but also secondary effects on social functioning, by interfering with children’s ability to participate in age-appropriate activities with peers (such as team sports).

The embodied theory of cognition posits that we should consider cognition in terms of its function in serving adaptive behavior (23). It follows therefore that complex adaptive behaviors, such as those of the hands, should be more closely related to cognitive functions than simple adaptive behaviors, such as those of walking. Reaching and grasping is a complex and fundamental motor activity that serves as a vital mode of exploration for children as they learn about the physical world (24). The ability to plan, execute, and monitor ongoing movement is an important aspect of completing an action toward a goal that is integrated in the environment (25). As such, disturbances in grasping patterns may impact how children play, explore, use tools, and engage socially. This review is aimed at providing a detailed summary of current knowledge of skilled use of the hands in ASD, focusing specifically on reaching and grasping.

**Figure 1:**
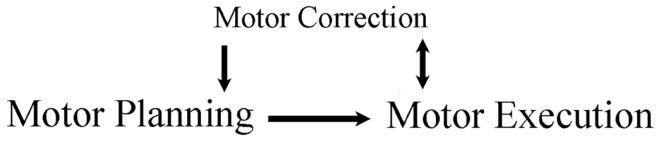
**Framework of review**. A movement is planned and then executed. The executed movement is monitored, as online corrections aide ongoing movements and offline corrections aide the planning of subsequent movements.

Systematic searches were performed in four computerized bibliographic databases (PUBMED, ISI WEB of Science, PsycINFO, ScienceDirect) to identify existing literature on reaching and grasping in ASD. The search terms included “reaching” and/or “grasping” with “autism.” Additional articles of interest were located in the reference sections of the articles from the systematic search. To be included in the review, a paper had to: (1) examine hand movements during reaching and/or grasping tasks in children with ASD, (2) include a comparison group of typically developing (TD) children (without a family history of ASD), but it could also include other groups for comparison, such as children with other forms of developmental disability (DD), and (3) confirm the ASD diagnosis using a multidisciplinary approach. Twenty-five articles met inclusion criteria and were included in the body of the review. The results of the search are presented below; beginning with a description of how manual-motor behavior develops in the first years of life in infants at-risk for, or diagnosed with, ASD. The remainder of the review is organized around the framework of a motor episode; describing how ASD affects motor planning and motor execution, as well as how ASD affects ongoing motor adjustment and knowledge across the lifespan (Figure 1). The review ends with a discussion of the implications of impairments to motor behavior, and how they relate to diagnosing ASD.

## Review Findings

### Early motor development

The analysis of early object manipulation may yield information on atypical development even before the onset of more core symptoms of ASD. During typical development, infants grasp objects and manipulate them using their oral, tactile, and visual senses to explore object characteristics (26). These sensorimotor skills are strongly associated with hand and finger sophistication in later development (27, 28). For example, after grasping a block, infants will bring it to their face to look at it, will rub their fingers along it to feel its texture, and will place it in their mouth to taste it. Atypical object exploration has been reported for infants as young as 12 months of age, who are later diagnosed with ASD. Compared with TD peers, infants who later received a diagnosis of ASD demonstrated more spinning and rotating of objects, as well as unusual visual exploration of objects (29). Retrospective parent reports of oral- and manual-motor skills from primary caregivers of children with ASD (*n* = 172) and TD children (*n* = 44) suggest that impaired oral-motor abilities (e.g., blowing a raspberry, sticking out tongue, and making animal sounds) and manual-motor abilities (e.g., grabbing dangling toys, block play) were able to distinguish ASD from TD children during infancy and toddlerhood (with sensitivity at 83% and specificity at 93% for oral-motor abilities and sensitivity at 89% and specificity at 86% for manual-motor skills in children later diagnosed with ASD). Surprisingly, correlational analyses revealed that oral- and manual-motor abilities of infants with ASD were better able to distinguish children with ASD from their TD peers than delays in the prototypical milestones of crawling or responding to name (30). A comparison of ASD and DD is necessary to separate the ASD-specific impairments from general delay when examining the associations between oral- and manual-motor abilities and social-communication outcomes. Nevertheless, oral- and manual-motor skills are not purely a “motor problem” and children with high verbal skills likely have better comprehension as well as expression, although such tasks do not require much verbal instruction.

Accordingly, several groups have examined whether oral, manual, and motor skills are related to diagnostic outcomes in infants at high-risk (HR) for ASD (for example, younger siblings of a child with ASD). Bhat et al. (31) examined the relationship between early gross motor behavior, as measured by the Alberta Infant Motor Scale [AIMS; (32)] at 3 and 6 months of age, and communication outcomes, as measured by the Mullen Scale of Early Learning [MSEL; (33)] at 18 months of age in HR (*n* = 24; 12 male) and TD infants (*n* = 24; 9 male). Compared to TD controls, HR siblings displayed the delayed motor performance on the AIMS at 3 and 6 months of age, but more importantly, all HR siblings who met criteria for a communication delay at 18 months of age exhibited a motor delay at 3 months of age. Mulligan and White (34) prospectively examined the relationship between sensory and motor behaviors in HR infants (*n* = 13; mean age 12.6 months; 5 males; 4 of the 13 were diagnosed with ASD at 30 month follow-up) and their TD peers (*n* = 12; mean age 12.1 months; 5 males) by asking infants and caregivers to participate in a 10-min play session and a 5-min eating session. Their behaviors were video-recorded and coded for the presence or absence of mouthing objects, object manipulation, hand to mouth with spoon, and plays with food. HR and TD infants showed a similar performance across the two sessions, although the HR infants moved around less and manipulated objects in their hands less frequently than the TD controls.

The relationship between poor motor ability and ASD continues into childhood. Using Part I (oral-motor assessment) of the Kaufman Speech Praxis Test for Children (35), Adams (36) compared oral-motor abilities and simple and complex phonemic production in children with ASD (*n* = 4; mean age 8.5 years) against a TD control group (*n* = 4; mean age 9.0 years). Children were asked to execute non-speech motor movements (e.g., pucker lips), produce simple phonemes (e.g., vowel-to-vowel movements), and produce complex phonemes (complex consonant production, polysyllabic synthesis). Children with ASD were impaired on performance of oral movements, particularly those involving in the tongue and lips, and these impairments impacted their ability to perform complex phonemic production and sound blending. In accordance with these results, Gernsbacher et al. (30) found that performance of oral- and manual-motor behaviors in ASD differed depending on level of verbal fluency. Minimally fluent (*n* = 20; mean age 7.4 years) and highly fluent children with ASD (*n* = 20; mean age 8.3 years) completed Part I of the Kaufman Speech Praxis Test for Children (35) and were coded as “able” or “unable” to complete tasks of “control saliva,” “protrude tongue,” “produce vocalizations,” and “pucker lips,” etc. Overall, the minimally fluent children were less able to complete oral–manual skills than the highly verbal children, showing impairment on tasks such as “open mouth,” “spread lips,” and any tasks involving with the tongue. Results such as these highlight the important relationship between non-vocal oral abilities and vocal production. An understanding of these impairments is important when assessing social and communication ability in HR infants, as well as older children with ASD, as impairments in oral- and manual-motor ability can confound the assessment of both verbal and non-verbal language, extending into the ability to engage socially with peers. That said, it is important to acknowledge that many factors contribute to communication functioning other than oral–motor skills. Moreover, difficulties comprehending instructions may confound assessment of motor skills in children with ASD who have receptive language delays, which may need to be taken into account in interpreting other findings summarized in this review.

### Motor planning

The analysis of motor planning may yield early information concerning impairments in cognitive processing in ASD (37). Before completing a motor act, such as reaching for a block to build a tower, a motor plan first needs to be developed. Motor planning involves the sequence of motor commands that convert the current state of one’s body into the desired state. Thus, when building a tower, a person must formulate a plan that consists of lifting his/her hand, extending it toward a block, shaping his/her digits to grasp the block, and then transporting the block to the table to begin construction.

#### Reaction time tasks

Recording reaction time is the simplest way to measure motor planning, as it provides a basic measure of the time taken to formulate a motor plan. The majority of studies report that participants with ASD typically show longer reaction times than their TD peers (18, 38–41). However, when presented with simple tasks, such as drawing a line between the two targets, children with ASD and TD perform similarly. Dowd et al. (42) investigated motor planning and motor execution in young children with ASD (*N* = 11; mean age = 6.2 years) and TD children (*N* = 12; mean age = 6.6 years) using a point-to-point movement task, in which participants were required to use a stylus to move between two points on a digital screen. Overall, ASD and TD groups did not differ on any measures examined, but the ASD group did have more variable reaction times. In a similar experiment, Papadopoulos et al. (43) presented adolescents with Asperger’s disorder (*N* = 20; mean age 9.6 years), high-functioning ASD (*N* = 19; mean age 9.8 years), and TD children (*N* = 18; mean age 9.8 years) with visual stimuli on a tablet; two small or large yellow circles were positioned on a horizontal plane from left to right and were separated by a space of 12–25 cm. The participants were asked to draw a line between the two targets as fast and accurately as possible. Kinematic analysis showed that time to complete the movement did not differ between the three groups; however, the high-functioning ASD group had more variable end-points when compared to the TD group, suggesting the lack of a well-formed movement plan following a series of repetitions. It is interesting that more variable reaction times are typical of children with Attention Deficit Hyperactivity Disorder [ADHD; (44)], and given that a substantial proportion of children with ASD also show signs of ADHD (45), it may be important to determine the specificity of the finding of high variability to ASD (i.e., examine children with ASD who do and do not show signs of ADHD).

When presented with a more complex task, group differences begin to emerge in relation to planning a movement. Glazebrook et al. (46) asked the participants with ASD (*n* = 9; mean age 26.9 years) and their TD peers (*n* = 9; mean age 25.1 years) to move their index finger as quickly as possible to an illuminated circular target after a starting cue. During the trials, the size of the targets as well as the distance between the targets varied. As reported with simpler tasks, adults with ASD had more variable performance than the TD controls, but they also required more time to prepare and execute their movements, and reached lower peak acceleration and velocity than TD controls. In a follow-up experiment, Glazebrook et al. (38) used a more complex experimental set-up consisting of a black box with 10 switches, 2 of which served as a start position for the index finger of each hand. Adults with ASD (*n* = 18; mean age 23.7 years; 17 male) and TD controls (*n* = 18; mean age 20.6 years; 12 male) were presented with a valid precue to indicate either hand required (left/right) or distance of the target to grasp (near/far). Following illumination of the target, the ASD participants took longer to respond and complete the movement, and again were more variable in responding than the TD controls. When performing the same task, but receiving an invalid precue, Nazarali et al. (40) found that adults with ASD (*n* = 12; mean age 26.2 years; 12 male) take longer to reprogram and complete their movement (as indicated by increased reaction and execution times) than their TD peers (*n* = 12; mean age 22.8 years; 10 male). The effect was even more pronounced for invalid “hand” cues than invalid “direction” cues. These results are of particular importance for planning deficits in ASD. That is, when presented with an invalid “hand” precue, additional sequences of movements must be included in the new plan (i.e., put down left hand, lift right hand, reach to left space), than if presented with an invalid “direction” cue (i.e., move left hand to left space instead of right space). It follows therefore that if ASD is indeed associated with a planning deficit, it would not be surprising that the ASD group would be more affected than their TD peers. In accordance, the complex tasks presented above require multi-level processing; seeing a cue, formulating a plan, and initiating a motor response. As such, it is possible that observed impairments on such tasks may not be purely related to motor skills *per se*, but rather from an incoordination between cognitive processing and motor output.

#### Reach and grasp tasks

That individuals with ASD take longer to respond to an invalid cue may lend further weight to findings from sequential motor tasks, which indicate that children with ASD may be less responsive to visual information when planning a sequential task. Using a reach, grasp, and place paradigm, Fabbri-Destro et al. (47) examined how children with high-functioning ASD (*n* = 12; mean age = 10 years) and sex and age-matched controls execute motor plans by manipulating the size of the container into which a grasped object is to be placed. While TD participants adjust the temporal characteristics of the reach and grasp components of the sequence based on the size of the final placement container, children with ASD did not alter how the movements were executed. The authors suggested that children with ASD program sequential movements in independent steps, rather than as a cohesive pattern and do not utilize the visual feedback of end-point target when planning their overall movement. Thus, it could be argued that the delayed response following the presentation of an invalid cue may not be due to planning deficits *per se*, but rather an impairment in registering and responding to visual feedback. Indeed, evidence from functional imaging of connective networking in the brain suggests that individuals with ASD have impaired communication between brain networks, and thus may have trouble coordinating a movement in response to a visual cue (48).

**Figure 2:**
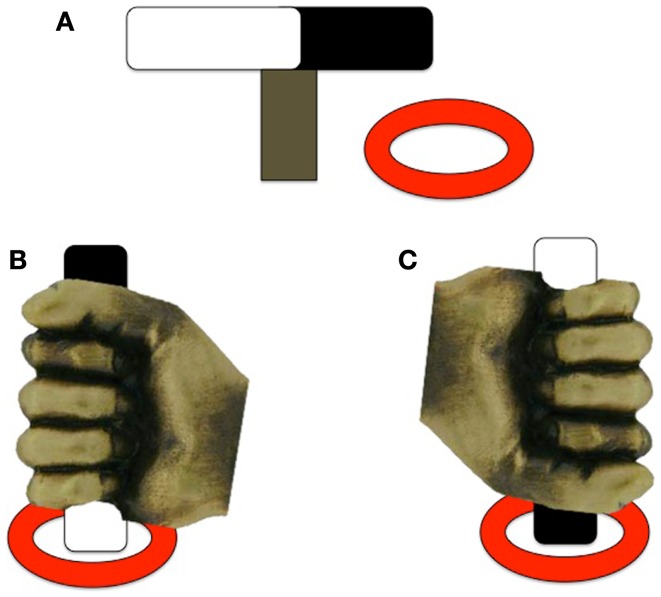
**Experimental design of the underhand grasps used in Hughes (17)**. **(A)** The rod and ring set-up; **(B)** example of a *comfortable* end-state underhand grasp; **(C**) example of an *uncomfortable* end-state underhand grasp. Note that the rod is positioned the same for each trial, only the color of the end of the rod to be placed in the ring differs between trials [adapted from Ref. (17)].

Hughes (17) examined motor planning in children with ASD by employing a reach-to-grasp task that encouraged a particular hand posture. Hughes also included a group of children with DD as a comparison group to help identify ASD-specific impairments to planning ability. Children with ASD (*n* = 36; 12–14 years), DD (*n* = 24; 10–12 years), and TD (*n* = 28; 3–5 years) were asked to pick up a rod that had one end painted black and the other end painted white and place one of the colored ends into one of two disks so that the rod stood upright. By varying the starting position of the bar, it is possible to encourage the participants to produce an overhand or underhand grip, leading to either comfortable or awkward final posture depending on their planning abilities (see Figure 2). The criterion for a correct response, and thus appropriate motor planning, was an appropriate hand action on the underhand trials, in which the person begins with an uncomfortable grasp to end with a comfortable grasp. There were no group differences on the overhand trials, which required no special planning (grasp horizontal bar and supinate wrist to place end closest to pinky finger into ring). For the underhand (uncomfortable) condition, however, the ASD group made fewer correct initial postures than the DD group, and both groups together performed more poorly than the TD group. Hughes (17) suggested that performance of the ASD group resulted from a fundamental deficit in motor planning leading to inability to plan a series of movements that would result in a comfortable end-grasp posture. However, a similar experiment using an end-state comfort task by van Swieten et al. (49) failed to detect motor planning differences between ASD and TD groups. Children with ASD (*n* = 20; age range 9–14 years), developmental coordination disorder (DCD; *n* = 11; age range 9–13 years), and TD peers (*n* = 44; age range 9–14 years) were presented with a wooden dowel attached to a rotating platform. One end of the dowel was painted red and the participants were told to place their thumb on the red end of the dowel as the start position, and rotate their wrist to move the dowel 180° to the end position. The children had to choose between performing either the minimum amount of rotation or end-state comfort (on 50% of the trials, these coincided). Interestingly, the ASD and TD groups performed similarly on the task, choosing end-state comfort on approximately 75% of trials; however, both groups differed from the DCD group, who more often chose minimal rotation over end-state comfort (approximately 60% of trials). The discrepancy between the findings from Hughes (17) and van Swieten et al. (49) may be due to the complexity of the plan required to complete the tasks. The Hughes (17) task parameters required the processing of three sequential aspects of the reaching motion; that is, participants needed to choose between an overhand and underhand grasp, lift the object, and either supinate or pronate their wrist to place the object in a hole. In contrast, the task of van Swieten et al. (49) only required the child to process one aspect of the motion (either supinate or rotate their wrist), begging the question of whether the motor impairments seen on the Hughes (17) task may be due to problems processing multiple pieces of information to formulate a succinct motor plan.

To summarize, analysis of motor planning in ASD has suggested increased variability in movement onset and offset (42, 43), increased reaction time to valid cueing (38, 39), delays in reinitiating and completing a movement following invalid cueing (38, 40), and impairments when planning a comfortable end-grasp posture, depending on the complexity of the plan required (17, 49). When taken together, the results of motor planning literature suggest that individuals with ASD have trouble in formulating a motor plan when asked to process multiple pieces of information (i.e., complex task), which may be cognitively taxing and thus interferes with motor output.

### Motor execution

Analyses of motor execution (that is, acts of carrying out planned movements) provide the opportunity to understand the neurological underpinnings of cognitive processing that precede such movements. Commands from the motor cortex are sent to the corresponding nerves and muscles to carry out the motor act. For example, after planning to grasp a block with the right hand, a person must then specify the particular muscle contractions to move the limb in the correct direction and shape the digits appropriately for grasping. Due to the reciprocal interactions between motor cortex, sensory input, and motor output, there are ample opportunities for errors to occur when executing a motor plan. Here, we review motor execution in children with ASD.

#### Grasping tasks

**Figure 3:**
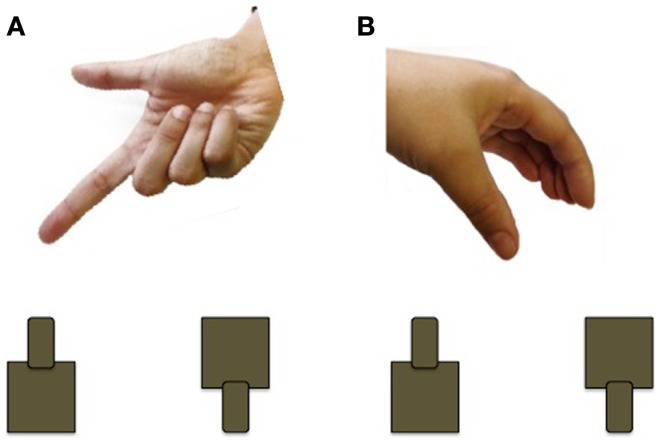
**Example of the cueing used by Stoit et al. (51)**. **(A)** Pointing cue to indicate cylinder to be grasped (left or right); **(B)** hand shape cue to indicate cylinder to be grasped (precision or power).

Using a grasp and place task, Forti et al. (50) found that the movement duration of participants with ASD is nearly twice as long as those of controls. Participants with ASD (*n* = 12; mean age 3.5 years; nine males) and age and sex-matched TD controls were instructed to transport a rubber ball from a start location and drop it into a hole, located 30 cm away, while wearing kinematic markers (markers placed on the body that allow the online/offline tracking of body segments). In addition to taking longer to complete the movement, children with ASD had higher velocities at movement terminus. Although the ASD group was able to accurately transport the ball and drop it into the hole, every member of the ASD group made corrections *at least once* after entering the area of the hole, whereas *fewer than half* the TD controls made corrections. Interestingly, there were no differences observed for the initial movement phases, which should reflect motor planning processes. In a related study, Stoit et al. (51) examined feed-forward motor control in children with ASD (*n* = 31; mean age 11.6 years) and TD children (*n* = 29; mean age 10.5 years) using a precision versus power grasp task. Feed-forward movements rely on internal models for accuracy and do not require the online use of sensory feedback evolving during the action (52). Participants were seated behind a table and presented with two cylinders, a small cylinder affording precision grasping and a large cylinder affording power grasping. For each trial, participants received a cue, administered by a human hand, to indicate the location (left/right) or grip-type (precision/power) of the target to be grasped (see Figure 3). As in the previous study, movement times were significantly longer in the ASD group, although there were no differences in initiation errors or time to respond following start cue between the two groups. Using a similar reach-to-grasp task, Mari et al. (18) report that reaching and grasping kinematics are largely uncoupled and executed in a successive non-overlapping manner in children with ASD. Children with ASD and their TD peers (*n* = 20 per group; 7–13 years) grasped wooden blocks of varying sizes and distances and specific kinematic measures were recorded, including time to reach peak velocity, deceleration time, as well as the coordination of the reach and grasp components. Because the reach component is controlled by the proximal musculature of the shoulder and elbow and the grasp component is controlled by distal musculature of the forearm and hand, it is possible that the ASD group might show an impairment of coordination. Overall, the children with ASD performed the movement quite well, and did not differ from their TD peers. Exploring the results further, the performance of the ASD group was contrasted by IQ. An identified “lower functioning” group (IQ range 70–79) showed evidence of desynchronization between the reach and grasp components, whereas the identified “higher functioning” group (IQ range 80–109) demonstrated a closely integrated and overlapping movement. These results highlight the importance of including IQ and/or developmental matched controls to determine specificity of findings to ASD.

The results of Cattaneo et al. (53) also support the incoordination of motor components of a reaching-to-grasp movement in ASD. Electromyography (EMG) recorded muscle activity related to mouth opening during an eating task in children with ASD and age-matched TD controls (*n* = 8; mean age = 6.5 years for both groups) showed that EMG activity started before the hand even grasped the object for the TD group. In contrast, EMG activity in the children with ASD started much later, when the hand was bringing the food to the mouth. A recent report by Pascolo and Cattarinussi (54) critically evaluated the results of Cattaneo et al. (53) and failed to replicate their finding of impaired synchronization between grasping and eating. Pascolo et al. employed the same methodology as Cattaneo et al. but applied increased control over the experimental set-up. For example, the supplementary information that accompanied the original article by Cattaneo et al. acknowledged that the distance between the child and the food varied across trials and there were extra personnel in the room when the experiment was conducted (which could be distracting). To examine the effect of these limitations on mouth activation, Pascolo et al. varied the distance of target (near, far, and comfortable distance) and had the children reach for food in a quiet room without extra personnel. Pascolo et al. (54) did not find any differences between the performance of the ASD group (*n* = 7; mean age = 7.3 years) and their TD peers (*n* = 12; mean age = 7.7 years), as both groups opened their mouth after the food had been grasped. Interestingly, when looking at the effect of distance on mouth opening, Pascolo et al. found that the further the target was away from the body, the later the onset of mouth opening. The lack of replication between Cattaneo et al. and Pascolo et al. likely relates to differences in experimental methodology employed. Pascolo et al. carefully controlled for two extraneous influences on the performance of children with and without ASD, by having them repeat the same movement numerous times in a quiet setting. Cattaneo et al. had children with and without ASD perform a grasping and eating movement in a more naturalistic setting, with variance in food location and extraneous persons present. The difference in set-up between these two experiments emphasizes the importance of task boundaries when considering experimental results. When presented with a quiet environment in which one movement is repeated, ASD children perform similarly to TD children. When they are presented with a more naturalistic environment, in which variance occurs between trials, and extraneous personnel are present, the cognitive system of children with ASD becomes taxed, resulting in impaired motor performance. This is in accordance with results from motor planning, which suggest that motor performance of individuals with ASD similarly become impaired when asked to process multiple pieces of information (17, 38, 47).

### Motor correction

The analysis of motor corrections can provide information on an individuals’ ability to understand and respond to ongoing stimuli in the environment. Ongoing adjustment during movement execution is termed *online control*. The internal representation of the movement is compared to the executed movement, and adjustments to the movement are made based on visual and proprioceptive feedback (55). For example, when lifting a block to build a tower, somatosensory feedback guides the application of differential force to blocks made of foam versus those made of wood.

#### Load-lifting tasks

In a unique experiment, Schmitz et al. (25) investigated motor adjustment during a bimanual load-lifting task in children with ASD (*n* = 8; mean age 7.9 years, six males) and their TD peers (*n* = 16; mean age 6.0 years; seven males). Participants wore a bracelet on their right hand equipped with a strain gage that supported a platform on which a load could be placed. Motor adjustment was measured from the angular displacement of the forearm along the elbow joint, and activity of bicep and tricep muscles were recorded using surface electrodes. The response of the arm was measured when the load was removed by the experimenter or by the participants’ left hand. The results indicated that the maximal angular amplitude of the elbow did not differ between ASD and TD children in either the experimenter- or self-unload conditions, although the latency for bicep inhibition took longer in the ASD group. The delay in bicep response of the ASD group suggests a lack of anticipation of the unloading force, and as such, they respond only after receiving sensory feedback that the load had been lifted from the platform.

A recent experiment by David et al. (24) examining motor adjustment included a comparison group of children diagnosed with DD to help distinguish between impairments due to general delay versus those that were ASD specific. Grip and load force were measured in children with ASD, DD, and TD peers (*n* = 21 per group; 2–6 years) during a grasp and place task. Grip force was measured from the digits on the grasping hand and load force was measured from the proximal musculature of the reaching arm and shoulder. Within the TD group, age was inversely related to grip-to-load force onset latency and time to peak grip force; however, there were no similar age-related decreases between grip and load force for either the ASD or DD groups, suggesting that the impairments to motor adjustment on this task reflect a maturational delay, rather than an ASD-specific delay. In an earlier report, David et al. (56) examined grip and load force adjustments in a group of older, high-functioning children with ASD (*n* = 13; mean age 11.2 years). The adolescents were instructed to lift a target from a start position on load cell and place it on a target platform, approximately 6″ away. On this task, the ASD group had longer grip-to-load force onset latencies, greater grip force at movement onset, and more variable performance than TD controls. However, peak grip force and time to peak grip force did not differ between the ASD and TD groups. The children with ASD consistently did not respond until the load was removed, suggesting they were unable to use ongoing experience to anticipate upcoming unload force.

#### Adaptation tasks

Motor adaptation is the modification of a voluntary movement based on error feedback between repeated trials (57). To be considered “adaptation,” the movement must change in respect to one or more parameters (e.g., force or direction), the change must occur gradually (i.e., over minutes to hours), and once these changes have occurred, the person must show “after-effects” and “de-adapt” the movement in a similar manner to return back to the original state (58). To understand the role of visual and proprioceptive feedback in motor adaptation in children with ASD, Masterton and Biederman (59) trained children with ASD (*n* = 11; mean age 10.4 years) and intellectual disability (ID; *n* = 11; mean age 11.6 years), as well as younger (*n* = 11; mean age 9.1 years) and older TD children (*n* = 11; mean age 14.1 years) to place a wooden block onto a target while viewing the target apparatus through a prism lens that displaced vision of their environment. Overall, the ASD and ID groups took longer to adjust their movements under the adaptation task, requiring almost double the amount of time to adapt to reaching with the prism glasses than both TD groups. Interestingly, transfer of motor adaptation of the reaching hand to the non-adapted (non-reaching) hand was found only for the ASD group. The authors suggest that the transfer of adaptation to the non-reaching hand is a clear indication that ASD children rely on proprioceptive, rather than visual information to complete the target-reaching task. It is possible that difficulty with processing sequential visual information may account for the ASD participants’ motor execution impairments and consequent reliance on proprioceptive input.

Other experiments examining motor adaptation have not reported differences in adaptation rates between ASD and TD groups. Gidley Larson et al. (60) had high-functioning ASD (*n* = 20; mean age 10.9 years; 17 males) and TD (*n* = 16; mean age 10.8 years; 11 males) participants complete a ball-throwing task at baseline without prisms (pre-adaptation), while wearing prism goggles (adaptation), and again without prism glasses (post-adaptation). In contrast to the findings of Masterton and Biederman (59), the ASD and TD groups showed similar adaptation rates and adaptation effects on movement performance. With a sub-set of the same participants, Gidley Larson et al. (60) further explored adaption in ASD by asking participants to grasp the handle of a robot tool to move a cursor onto a target, which was presented on a screen. The view of the hand controlling the robot tool was blocked throughout the task. On some of the trials, a perturbation (force or visual) was given to assess for participants ability to plan alternate strategies. All children exhibited clear indications of adaptation and reached similar rates of adaptation to the force and visual perturbations, with no significant group differences on any of the measures. The discrepancy in findings may result from the simpler adaptation tasks in Gidley Larson et al. (60) (i.e., throwing a ball and moving a robot tool), compared to those of Masterton and Biederman (59), which required the grasping and placement of small blocks, a more cognitively taxing task.

#### Motor knowledge

The ability to calibrate our body to perform motor actions is referred to as affordance perceptions. When shaping our digits to grasp, we use a smaller aperture for a block to be obtained with a pincer grasp and a larger aperture for a block to be obtained with a power grasp when building our tower. Affordance perception contributes to successful performance of many motor and non-verbal social capabilities. For example, when participating in team sports, such as badminton, one needs to be able to calibrate his/her body to hit the shuttlecock lightly, compared to tennis, in which the ball needs to be hit with more force. Being able to adjust one’s body allows for successful motor performance on both tasks. Linkenauger et al. (61) determined that adolescents with ASD poorly estimate their motor affordances when presented with a perceptual-motor integration task. Youth (*n* = 12; mean age 11.1 years; all male) and adults with ASD (*n* = 8; mean age 22.4 years; all male), and age- and sex-matched TD controls were asked to estimate the maximum extension of their reaching arm (i.e., how far they could reach), as well as maximum digit aperture (i.e., the largest foam block their digits were able to grasp). Following their estimates, participants completed a reach distance task and grasping task to determine their maximal actual values. The ASD groups made drastically larger errors (17–20% for youth; 14–26% for adults) than the TD groups (3–5% for youth; 5–7% for adults), suggesting they overestimated their motor affordance. These findings raise the possibility that motor deficits in ASD could originate in the inability to use the motor system to determine action capabilities and utilize prior knowledge of our own capabilities to aid in planning and executing the task at hand.

To examine the relationship between action understanding and ASD, Cossu et al. (62) presented high-functioning ASD children (*n* = 15; mean age 8.1 years; 13 males) and two TD samples, one matched for chronological age (mean age was 8.7 years) and a second, for younger chronological age (mean age 4.9 years), with three tasks. The children watched a video clip and were asked to imitate actions (conventional or non-conventional actions on objects), produce pantomimes of actions (e.g., shown a tool and required to pantomime the correct action of the tool), or understand a pantomimed action (e.g., watch an actor mime an action without an object and point to the object “used” in the pantomimed action). The authors found that the children with ASD were significantly worse at imitating conventional actions on objects, imitating finger posturing, and imitating oral–facial gestures than both the younger and age-matched controls. The children with ASD performed similarly to the younger control group when identifying tools used in pantomimed actions, but both groups performed worse than the older TD group. The simultaneous impairment of action imitation, production, and comprehension of pantomime action suggests that the process of constructing an action motor representation is impaired in children with ASD. Critically however, is that language ability was not controlled for in these studies. It has been reported that the ability to imitate familiar gestures (such as conventional actions on objects) is correlated with language comprehension (63). Without controlling for language ability, one cannot rule out that the lack of imitation may be the result of reduced comprehension of the task requirements (64).

In summary, individuals with ASD appear to be impaired in both the online [i.e., use of ongoing sensory feedback; (25)] and offline control of movement [i.e., using memory from previous trials; (24, 56)], as well as in estimating their motor abilities (61). That is, they are unable to use both ongoing visual feedback, as well as information from a previous movement, to plan subsequent movements more effectively [also noted by Khan et al. (65)]. These impairments may result from deficits in the visual control of movement in ASD, resulting in an increased reliance on proprioceptive feedback to complete movements [as supported by adaptation transferring to the non-adapted hand for ASD only; (59)].

## Discussion

There is now robust evidence from early motor development, motor planning and execution, as well as motor correction that movement is impaired in ASD. Very young children display abnormal play with toys (e.g., spinning, flicking), less toy play, and atypically visually explore objects (29, 30). As they get older, children with ASD show impairments in motor planning, including delays in movement initiation and impairments when planning complex sequences of movements resulting in a comfortable end-grasp posture (17, 38, 42, 43), and impairments in motor execution, such as increased movement duration, end-point corrections at movement terminus, and desynchronization between components of a reaching movement (38–40, 50, 53). Impairments to online and offline corrections are also evident, as they are unable to use both ongoing visual feedback, as well as information from a previous movement to plan subsequent movements more effectively (24, 25, 56). One might postulate that abnormal toy play, including abnormal sensory control, in very young infants could interfere with subsequent opportunities for motor learning and may also impact social communication. For example, if a child has trouble in grasping an object, and continues to stare at it as he or she spins the object in his or her hands [as per Ref. (29)], the child in turn may spend less time showing the object to a parent or friend and engaging in other joint attention behaviors.

Are motor impairments and cognitive outcomes in ASD related? Findings linking motor ability to outcomes in individuals with ASD have been replicated numerously in the literature (66–68). For example, the transition to independent sitting is associated with greater variations in babbling (69), motor delays at 18 months of age are highly predictive of a diagnosis of ASD at 3 years of age in HR toddlers (70), and better motor performance in newly diagnosed 2-year-olds with ASD is associated with better future outcomes at 4 years of age (71). Although delays in motor and communication development may represent co-existing but relatively independent aspects of the ASD phenotype, there may be consequences of motor delays that impact on opportunities for developing and practicing social-communication skills. For example, if a child is delayed in sitting, and spends most of his or her time on the tummy, then he or she would have less time with the hands free to engage in reaching and grasping for objects, showing objects, and requesting objects than an infant who has matured to a sitting position. As such, the onset of these “social” behaviors may also be delayed. This is consistent with the findings of Libertus and Needham (72), who found that TD infants who engage in active, self-produced reaching movements also engage in spontaneous orienting to faces, whereas infants who engage in passive toy play (watching others play with objects) showed less spontaneous orienting to faces. Clearly, there are other factors that influence social-communication development, as well as examples of neurological conditions associated with severe motor impairments yet relatively preserved social skills [e.g., early onset neuromuscular disorders; (73)]. However, there is evidence that motor- and social-communication skills are correlated in ASD, both in the school age years (74) and in infancy (31). Moreover, gross and fine motor delays may be among the earliest identifiable signs distinguishing infants with ASD from their TD peers (75–77).

Impairment in object manipulation may also impact how others’ actions are understood (51, 78–81). Evidence for this comes from findings that, during action observation, mu rhythm desynchronization is less evident in ASD. Mu rhythm is a pattern of electrical activity that comes from the area of the brain that controls voluntary movement (primary motor cortex) when at rest. When large number of neurons synchronize in preparation for a movement, or when viewing an actor making a movement, the mu rhythm is described as “desynchronized” (82). Bernier et al. (83) found reduced mu rhythm desynchronization during movement observation in ASD, and reduced desynchronization was associated with poorer imitation skills. Similarly, Oberman et al. (84) report that, although individuals with ASD exhibit desynchronization of mu rhythm during voluntary movements, mu desynchronization is absent when observing an actor perform the same movement. Interestingly, the degree of mu desynchronization in ASD is sensitive to level of familiarity, only responding when individuals can identify with the stimuli in a personal way (85). The lack of a mu desynchronization response when observing an actor may result from an impaired mirror neuron mechanism (MNM) in ASD (62). Mirror neurons are involved in imitation of simple movements (86), learning of complex skills (87), in the perception of communicative actions (88), and in the detection of basic action intentions (89). Parietal mirror neurons code the goal of both an executed and observed motor act, such as grasping an object, and also code the overall intention of the action, whether the actor intends to bring the grasped object to the mouth or place it in a container (90–93). Deficits in the MNM have been reported during movement execution and observation for children with ASD [Ref. (94, 95); see review by Rizzolatti and Fabbri-Destro(96)]. As mentioned previously, Cattaneo et al. (53) employed EMG to record muscle activity related to mouth opening during an eating task in ASD. When observing an actor pick up a food item and transport it to the mouth, EMG increases in mouth muscles were found for the TD controls, but not for the ASD group. These results suggest that children with ASD have impaired mu desynchronization that may translate to a dysfunctional MNM. Such impairments may impact motor learning and action understanding, which may ultimately lead to misinterpretation of others’ actions.

Although mirror neurons play an important role in action execution and observation (97, 98), they are unlikely to fully account for the myriad of motor impairments displayed by individuals with ASD. Pathological studies consistently report abnormalities in brain regions known to mediate motor function, including the cerebellum and subcortical white matter (99–106). The cerebellum is one of the key structures required to form accurate internal models of motor acts, making reciprocal connections with motor areas of the cortex to carry out planned corrections during movement execution (107, 108). As such, it is likely that cerebellar abnormalities play a role in movement correction impairments seen in ASD, as well as impairments to eye movements [such as prolonged staring; for a recent review of the role of the cerebellum in ASD, see Ref. (109)]. In addition, abnormal connectivity between adjacent primary sensory and motor areas has been reported in ASD (48, 51), and may account for impairments seen during the online control of movement (110). Moreover, reduced connectivity between more distal areas of the motor system, such as between visual and motor regions sub-serving action, may be responsible for impairments in motor planning and motor execution in individuals with ASD (48, 51).

How do motor impairments relate to social impairments? Typically, a child has a full repertoire of movement that he or she can use to engage in social interactions. With respect to the current review, the ability to properly manipulate objects is important for activities of daily living (such as brushing teeth), engaging in solo play activities (such as assembling a puzzle), and participating in team sports (such as baseball). Yet, many children with ASD have impaired motor behavior, detectable as early as 3 months of age (31). Being able to participate in peer play would require a child to respond in a timely manner (catch a ball before it hits you or the ground), perform skilled motor tasks (hitting a ball with a baseball bat), engage in eye contact (to understand and show action intention), and respond to social cues (understanding when it is appropriate to steal a base). Many of these behaviors are those that are impaired by ASD. Not surprisingly, Leary and Hill (20) propose that motor ability might have a significant impact on the core characteristics of ASD. The idea is, when a person is unable to respond to another’s action in a timely fashion, he/she will miss the positive reinforcement associated with interpersonal interactions. A child’s experiences throughout development may be drastically altered if, at an early age, he/she is unable to remain involved in social interaction, and as a result, may withdrawal from social activities [reported in Ref. (20)]. This “motor cognition” perspective does not imply that social impairments are a direct result of motor impairments, but rather that impaired movements may interfere with opportunities for positive social experiences and thus, social learning. Conversely, reduced social interaction opportunities may also contribute to poor action understanding. Thus, the relationship between social and motor competencies/impairments may be reciprocal in ASD, a hypothesis that remains to be explored in future longitudinal research.

There are common methodological limitations present in the literature reviewed here. First, many of the articles have relatively small sample sizes; Adams (36) sample consisted of only 4 children with ASD, Glazebrook et al. (46) recruited only 9 children with ASD, and sample sizes of individuals with ASD in the other studies ranged from as few as 8 (53) to as many as 36 (17). With the small sample sizes in several of the studies, there is a risk of participation bias in oversampling individuals with ASD who present with motor difficulties. Second, there is quite an age range in several of the experiments. The developmental course of motor development in individuals with ASD is not well understood, particularly when considering the timespan from toddlerhood to adulthood. Because of this, it is difficult to compare the results from one age group (i.e., young childhood) to another (i.e., adulthood). Third, there is a general lack in appropriate controls. When determining the ASD-specific deficits in movement, many studies report the use of TD control only. However, the results of Hughes (17) and David et al. (24) highlight the importance of including a control for intellectual or developmental level. Similarly, the results of Mari et al. (18) demonstrate the importance of stratifying intelligence when interpreting experimental results. Fourth, importantly, the severity of ASD symptomology varies across the studies reviewed here, and as such, the comparability of study conclusions might be constrained by the methodological limitations present in the literature.

Overall, there has been much research examining the relationship between social communication and motor behavior in ASD. To fully engage in social interaction, a child has a full movement repertoire of functional actions for use in communication and for understanding the communicative nature of others’ movements (111). A shift in focus to this “motor cognition” perspective suggests that interventions for children with ASD should include both a motor and a social component, as there is ample evidence that impairments in cognitive function are associated with impairments in movement (70, 76, 112–115). Many activities that promote social skills, such as cooperative board games or card play that involve turn taking, require the use of fine motor skills (e.g., grasping small game pieces, shuffling cards). As such, incorporating motor training into intervention programs could boost confidence in action capabilities and promote socialization and communication.

## Authors Contribution

Lori-Ann R. Sacrey made substantial contributions to conception and design of the review, collected and reviews the papers, prepared the first draft of the paper, and approved the final draft. Tamara Germani contributed to the conception of the review, provided a critical review of the manuscript, and approved the final draft. Susan E. Bryson contributed to the conception of the review, provided a critical review of the manuscript, and approved the final draft. Lonnie Zwaigenbaum contributed to the conception of the review, provided a critical review of the manuscript, and approved the final draft.

## Conflict of Interest Statement

The authors declare that the research was conducted in the absence of any commercial or financial relationships that could be construed as a potential conflict of interest.
